# Behavioral Patterns of Supply and Demand Sides of Health Services for the Elderly in Sustainable Digital Transformation: A Mixed Methods Study

**DOI:** 10.3390/ijerph19138221

**Published:** 2022-07-05

**Authors:** Siyu Zhou, Ziling Ni, Atsushi Ogihara, Xiaohe Wang

**Affiliations:** 1 School of Public Health, Hangzhou Normal University, Yuhangtang St., Yuhang, Hangzhou 311121, China; siyuzhou@hznu.edu.cn (S.Z.); ziling@hznu.edu.cn (Z.N.); 2 Department of Health Sciences and Social Welfare, Faculty of Human Sciences, Waseda University, Tokorozawa 359-1162, Japan; aogi@waseda.jp

**Keywords:** sustainability, digital transformation, suitable for aging, service ecological theory, health service

## Abstract

The aging transformation of digital health services faces issues of how to distinguish influencing factors, redesign services, and effectively promote measures and policies. In this study, in-depth interviews were conducted, and grounded theory applied to open coding, main axis coding, and selective coding to form concepts and categories. Trajectory equifinality modeling clarified the evolution logic of digital transformation. Based on the theory of service ecology, a digital health service aging model was constructed from the “macro–medium–micro” stages and includes governance, service, and technology transformation paths. The macro stage relies on organizational elements to promote the institutionalization of management and guide the transformation of governance for value realization, including the construction of three categories: mechanism, indemnification, and decision-making. The meso stage relies on service elements to promote service design and realize service transformation that is suitable for aging design, including the construction of three categories: organization, resources, and processes. The micro stage relies on technical elements to practice experiencing humanization, including the construction of three categories: target, methods, and evaluation. These results deepen the understanding of the main behaviors and roles of macro-organizational, meso-service, and micro-technical elements in digital transformation practice and have positive significance for health administrative agencies to implement action strategies.

## 1. Introduction

Relying on the data gathered by recently developed information and communication technologies (ICT), studies on sustainable public services and social governance have entered a new era. The main goal of sustainable digital development is to promote the inclusive application of digital services in the fields of medical care, health care, and elderly care [1,2,3,4]. Improving the digital capabilities of social services and expanding multi-scenario applications in the digital society will promote the digital transformation of public services in the health field [5,6,7].

Digital transformation has facilitated the diversification of social structures, which has not only altered people’s lifestyles but also further promoted an integration of social and health care services [8,9]. The digital transformation appears to be a serious and growing public health problem, which seems to be a lot more present in the elderly compared to the younger population [10,11,12]. The elderly have generally been marginalized as digital users, which leads to problems such as “the inadequate digital literacy among the elderly have contributed to service underutilization”, “complex process have restricted the efficiency of health services”, and “the multiple digital tools reduced the sense of participation of the elderly” [13]. Various countries pay more attention to the health of the elderly, and several initiatives and policy interventions have been put in place to facilitate access and utilization of health care services by the elderly [14,15]. In China, the main model is to use digital technology to optimize service processes, guide the elderly to actively obtain health services, and improve the efficiency of health services for the elderly [16,17].

The cognitive decline of the elderly makes it difficult for them to integrate into the digital society, which has restricted their access to public services such as health in the digital environment. How to bridge the “digital divide” on the demand side of the elderly and redesign health services in a digital and age-appropriate manner from the supply side of public services has become an important theme in the field of social governance. Thus, this study will answer three questions: (1) what factors affect the aging-appropriate transformation of digital health services? (2) How can digital services be redesigned to fit the experience of the elderly and meet their health needs? (3) How can the government and social organizations take effective measures and strategies to provide the elderly with high-quality and convenient digital medical and health services?

To this end, this study firstly constructed a digital transformation framework for public services in the health field based on literature research. Through questionnaires and focus group interviews, the influencing factors of the health needs of the elderly under the digital transformation of public services were analyzed, and grounded theory and trajectory equifinality modeling methods were used to code and analyze the data obtained in the focus group interviews. Measures and strategies for digital transformation of elderly health services are proposed from three aspects: organizational, service, and technical elements.

## 2. Literature Review

To explore the framework of sustainable digital transformation of the elderly’ health services from the perspective of service design, this study mainly focused on theoretical literature regarding the elements of digital transformation of public services, the experience and service design path for the elderly, and the operational mechanism of digital transformation of public health services. The benefits of using digital health services for the elderly have been recognized by numerous studies. Regarding health management, the use of wearable devices assists them to increase their range of motion and reduce their fall risk [18]. Digital technology has also been shown to help them lose weight and prevent chronic diseases such as hypertension and diabetes [19]. In terms of mental health, the elderly’s use of digital technologies improves their self-efficacy [20], and the ability to connect with the outside world through digital media reduces loneliness [21]. However, they also face challenges with digital health services and some experience anxiety due to their inability to master the technologies [22]. In addition, data collection by digital technology has caused privacy anxiety [23].

### 2.1. The Elements of Digital Transformation on Public Services

Digital transformation in the field of public administration comprises mainly the transformation of government governance models. The specific measures of the model use digital technology to help the government obtain and transmit more data, information, and knowledge to achieve the goal of government governance and to extend the management hierarchy to the grass-roots organizations [24,25]. Technology-enabled models of care could improve the efficiency of health services, but patients’ sensory impairment can also lead to a weakening of the technical effect. Stakeholder research has suggested that health care providers have recognized that adopting, expanding, and sustaining technology-enabled health care models has generally benefitted patients, clinicians, and health services. These models require robust clinical trials and health service evaluations [26,27,28].

The digital transformation of public health services is divided into three aspects: medical institution service management, service process, and service technology tools. Medical institution service management means that health service personnel can realize the whole-process health service and management of patients by applying digital technology [29]. Service process means that patients can take the initiative to understand and give feedback on health services on the Internet [30]. Service technology tools refer to the interaction between doctors and patients using tools such as mobile phones or wearable devices [31]. The digitization of medical service management emphasizes that the hospital realizes the intervention of patients through information technology, and its mode is to use the Health Information System (HIS) system to achieve disease management [32]. The service process focuses on the “people-oriented” aspect of health human resources, requires the implementation of patient-focused chronic disease management, and its main model is family doctor health services [33]. Service technology tools focus on hardware configuration and use digital technology to interact with patients to achieve the goal of health management. Overall, this model uses digital tools as an intermediary tool for patient management [34].

Relying on the elements of “organization–service–technology” to build a digital transformation framework, this model implements service model reconstruction with patients as the core, emphasizes the provision of digital services from the supply side, and reduces digital adaptation barriers on the demand side. Among the three elements of digital transformation of public services, technical elements have been shown not to be one of the factors that lead to the dilemma of digitalization, while organizational elements and service elements are the key elements to respond to the real dilemma of the aging transformation of digital health services [35].

### 2.2. The Experience and Service Design Path for the Elderly

Service design is a human-centered mindset, a collaborative process of subjects, a set of experimental tools, an interdisciplinary language, and a diverse management approach [36]. The value creation of public services is an important issue in public administration. Public service design involves the connection and dynamic communication between public service users and organizations and is a process of creating value for users based on experience optimization [37]. Human-centered digital health design enhances the equity of health services. Concurrently, it can enhance the patient’s experience and help doctors obtain more data feedback, thereby helping health service providers to transform behaviors according to patients’ needs [38,39]. The design of digital health services for the elderly must focus on “designing for experience,” rather than simply adding digital technology to improve efficiency.

According to the matching of organizational and service elements in the context of digital transformation, the service ecosystem theory was used to construct a digital health service design for the elderly. A service ecosystem is “a relatively independent, self-adjusting system of resource integration actors that creates mutual value through shared systems and service exchange” [40]. In the elderly population, health policy, service design, and health service provision are the main factors affecting health levels [41]. The service ecosystem constitutes a key concept of the Service Dominant Logic, which defines actors as part of a larger system. This concept expresses that the activities of the elderly at the micro level are influenced by the activities at the meso and macro levels. The service ecosystem focuses on the micro level (experience in the life of the elderly), meso level (value realization of digital health service design), and macro level (institutionalization of digital health service design), which is the main path of public service design in the health field. Studies have found that remote communication through digital tools can effectively help the elderly reduce the risk of anxiety and depression [42]. Digital service design in the field of healthcare is one of the effective measures to improve the quality of life of older patients [43]. Furthermore, the institutionalization of digital technology can effectively broaden the social relations of the elderly [44].

### 2.3. The Operational Mechanism of Digital Transformation in Health Services

Digital transformation reconstructs the mode of social operation, releases the kinetic energy of digital society, and reflects the resilience of social operation [45]. In the process of digital transformation, it is necessary to grasp the coordinated development of the three elements of cyber–social–technical. The digital environment, composed of artificial intelligence and the Internet of Things, jointly promotes innovative ways of digital transformation [46]. Social factors, such as policy and education, can influence patients’ choice of using digital health services [47]. Digital technology realizes the health monitoring of patients through the network, thereby improving their self-health management ability [48]. The coordinated development of the three elements of cyber–social–technical will continue to expand the application scope of digital health [49]. Under the digital transformation of public health services, the elderly was prone to fall into the “digital dilemma,” which requires the neutrality of digital technology and the inclusion of humanistic transformation strategies regarding active aging and information accessibility [50]. The operation mechanism of digital transformation is designed from the supply side, including the transformation of the data governance, organizational, and service mechanism. The transformation of the data governance mechanism requires management agencies to apply digital architecture methods to optimize the allocation of human, financial, and material resources. Research on medical institutions often analyzes the impact of governance structure types and mechanisms on network effectiveness, emphasizing the contextual characteristics of network governance and effectiveness [51]. At the level of global health governance, national economic, democratic, and individual socioeconomic elements are multiple elements of a data governance mechanism [52]. The organizational mechanism must establish a flexible organizational structure that is streamlined, networked, and ecological. Another goal is to establish a data-driven dynamic optimization mechanism for an organizational structure. Digital technology affects the resiliency of health care at the organizational level of health management and organizational integration technology has become a driving force for the transformation of health services [53]. Especially in organizational management, digital technology is the main tool to rebuild the public service environment and is a mature practice field of digital technology [54]. The service mechanism requires the establishment of a service process and structure centered on the elderly to achieve people-oriented value benefits based on organizational and service elements. The service mechanism focuses on the design of specific service processes and provides health support to unequal and marginalized populations based on the use of digital technologies [55]. There are also significant differences between different regions in digital health care, and services must be designed according to local conditions [56]. The concept of “people-centeredness” as being the primary value of health services can help improve the patient participation rate in the service process [57].

**Figure 1:**
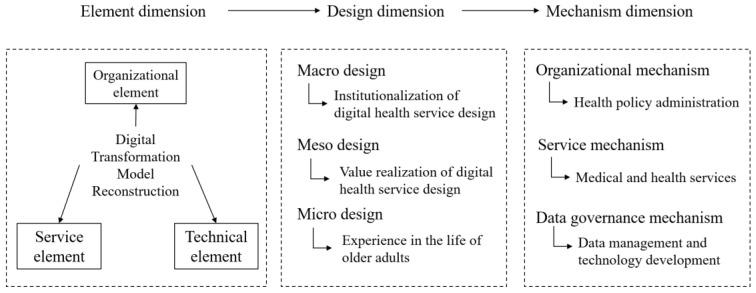
Framework for digital transformation of public health services.

In conclusion, the digital transformation of public services in the health field is presented in the element, design, and mechanism dimensions. The element dimension contains organizational, service, and technical elements; the design dimension includes macro, meso, and micro design; and the mechanism dimension includes the organizational, service, and data governance mechanisms. This framework is presented in Figure 1 below.

## 3. Materials and Methods

### 3.1. Participants

This study selected the Z District, Hangzhou City, Zhejiang Province as the research area and selected community managers, medical and health service workers, and the elderly in this area as the study subjects. The subjects were required to work or live in this district for more than five years and be deeply involved in the digital transformation of health services. The main reasons for choosing District Z as the research area were as follows: (1) District Z has undergone digital transformation earlier in Hangzhou and the digital transformation of health services began in 2014. The mature service foundation and transformation experience provided a research field for the extraction of digital health service transformation research. (2) District Z has the highest degree of aging in Hangzhou and there are many health service institutions in the area. Thus, there are many types of health services available to the elderly, which provides a foundation for research on digital health service experience. (3) In the process of digital transformation in the health field, the Z District has established a digital service platform in cooperation with health administrative departments, health service agencies, and health management companies. Based on the collaborative services carried out by multiple subjects, it provides a research distinction for the behavior changes of different subjects under the transformation mechanism; thus, has strong typicality and representation. The participants of this study were recruited in January 2021.

### 3.2. Research Method

The research on digital transformation of public services is a research hotspot in the field of public administration today. Thus, this study combined quantitative (questionnaire survey) and qualitative data (focus group interview) using a mixed-method approach.

The quantitative aspect aimed to determine the factors influencing the digital transformation of health services and the satisfaction with the digital transformation from the supply and demand sides. The questionnaire was divided into four sections: the first section investigated perceptions of the digital transformation of health services, the second section investigated the effect of the digital transformation of health services, the third section examined satisfaction with the digital transformation, and the fourth section collected the basic demographic information of the participants.

The qualitative aspect aimed to determine the architecture and path of the digital transformation of health services. There is a lack of mature research on the mechanism and effect of digital health services for aging transformation. Thus, this study conducted exploratory research using grounded theory and trajectory equifinality modeling. Grounded theory advocates that no assumptions are made in advance and the core concepts that reflect social phenomena are found on the basis of collected data. Furthermore, grounded theory forms theories by establishing connections between concepts. The goal is not to test theories, but to construct and develop them [58]. The trajectory equivalent modeling method belongs to the trajectory path balance model, which is a time series model used to record and analyze the behavior changes of the population over time in an irreversible time range. This method describes and builds a model of the diverse behaviors generated in the time-varying phase according to the specific experience of the population. Its main feature is that it can completely describe the change trajectories of various behaviors in a time series and can explain the causal path of behavior changes [59]. Trajectory equifinality modeling describes the influence of external forces, such as policy and society, on behavioral change processes to identify bifurcation points and iso-endpoints reached by research subjects. It illustrates the path of behavioral change due to the interaction of various forces. Primary directions are classified as behavioral leads (solid lines), indicating that a force tends to push toward the end. Non-primary directions (dashed lines) indicate that a force does not push towards the end [60]. The above two methods reflect the perceptual feedback and transition time series effects of the elderly population under the transition of digital health services to suit the aging process, dig deep into the elements that cause the transition dilemma, and analyze the causal logic behind this.

### 3.3. Quantitative Research Process

**Figure 2:**
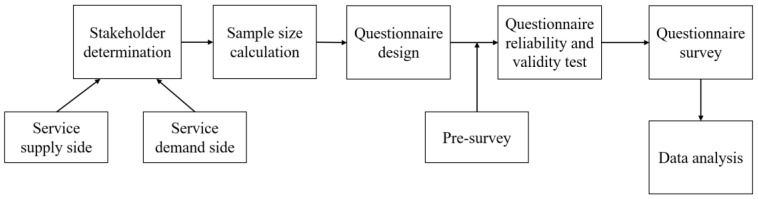
Research procedure of the quantitative research.

This research used a cross-sectional study of stakeholder questionnaires for digital transformation. The research procedure is shown in Figure 2.

#### 3.3.1. Participant Selection

Community managers, medical and health service workers, and the elderly in District Z were selected for the study. According to the sample size formula $N=Z^{2}\times \left(P\left(1-P\right)\right)/E^{2}$, the confidence level was set to 95%, *E* = 10%, *P* = 0.5, and the total sample size was at least 96 elderly. Considering that the sample should be representative, a cluster sample of community managers and health service workers was used. The community managers selected all the management personnel of the Z District, a total of 32 people. Health service workers selected all the medical staff of the Z District Community Health Service Center, a total of 34 people. There were a total of 66 supply-side personnel for digital transformation. Stratified sampling was used for the elderly, and 30 people aged 60–69, 30 people aged 70–79, and 30 people aged 80–89 were selected according to the electronic health record database. A total of 90 elderly on the demand side of digital transformation were included, with a total sample size of 156 people. All people provided informed consent and signed informed consent forms. The survey period for this study was March 2021.

#### 3.3.2. Data Collection Procedures

Quantitative data were collected through questionnaires. The questionnaire consisted of four parts:

1. Socio-demographic characteristics: including gender, age, living conditions, educational background, and health self-assessment of participants. Self-rated health ranged from 1 (very unhealthy) to 5 (very healthy).
2. Perception of digital transformation: it included the perception of organizations, services, and technologies under digital transformation. The items were: Your perception of the transformation of digital services in the organization; Your perception of digital health service projects; Your perception of technology in digital health services.
3. Identity of digital transformation: including the identity of the organizations, services, and technologies under digital transformation. The items were: Do you think digital transformation in the organization is effective; Do you think digital transformation in health services is effective; Do you think the application of digital technology is effective.
4. Satisfaction with digital transformation: it included satisfaction with organizations, services, and technologies under digital transformation. The items were: You are satisfied with the transformation of digital services in the organization; You are satisfied with the health services in digital transformation; You are satisfied with the application of technology in digital transformation.

The questionnaire consisted of three items: perception, identity, and satisfaction of digital transformation with a five-point Likert scale with scores ranging from 1 (strongly disagree) to 5 (strongly agree). The scores for the separate items were summed and divided by the total number of items. The formula was Score = $\sum_{i=n}^{n}\left(a+\beta \cdots +\delta \right)/n$.

Exploratory factor analysis was conducted to assess the nine items retained in the item analysis. The results demonstrated a Kaiser–Meyer–Olkin (KMO) test statistic for the evaluation questionnaire of 0.731. The factor analysis results after rotation are shown in Table 1. The reliability of the questionnaire was checked using Cronbach’s Alpha, and the reliability coefficient was 0.792.

#### 3.3.3. Statistical Analysis

All data analyses were conducted using Python (version 3.9.0, Python Software Foundation, Beaverton, OR, USA). Descriptive statistics, one-way analysis of variance (ANOVA), Pearson correlation analyses, and binary logistic regressions were employed. Perception, identity, and satisfaction were quantified and expressed as means (M) and standard deviations (SDs). The statistical analyses were performed using student’s *t*-test or one-way ANOVA, according to the characteristics of the data. The Pearson’s correlation coefficient (r) was used to assess the associations between variables. The strength of correlations was described as weak (|r| < 0.3), moderate (0.3 < |r| < 0.5), or strong (|r| > 0.70). To evaluate the perception, identity, and satisfaction of the organization, service, and technology, scores of >3 were classified as high, while scores of ≤3 were classified as low. Binary logistic regression was used to assess the effects of individual characteristics on the perception, identity, and satisfaction of digital transformation.

### 3.4. Qualitative Research Process

**Figure 3:**
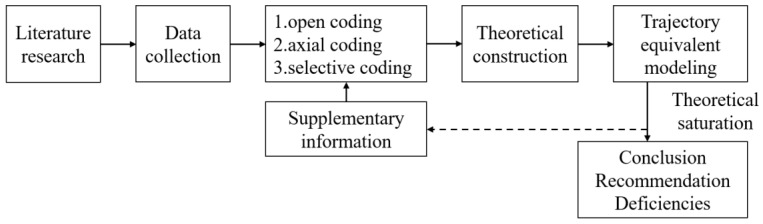
Research procedure of qualitative research.

This study was performed within the theoretical framework of grounded theory and trajectory equivalent modeling. The research procedure is presented in Figure 3.

#### 3.4.1. Participant Selection

The interview phase took place in April 2021 and June 2022. Interview data were generated via individual open-ended interviews and focus group interviews. Text data were obtained by sorting out management records and news reports. Semi-structured in-depth interviews and focus group interviews were arranged in separate offices in the community center and in university conference rooms, respectively. Considering that the participants included different age groups, our interviewer inclusion criteria were: (1) experienced in communicating with respondents of different age groups; (2) an ability to master the basic concepts and knowledge of digital transformation; (3) had basic knowledge of health services.

In the interviewee recruitment stage, to study the evaluation of digital transformation by different stakeholders, we randomly selected 20 digital health service providers in the community (including five community managers, five family doctors, three health nursing staff, three volunteers, and four technology developers) and 24 elderly (each from three different communities); thus, 44 people were interviewed. After obtaining the interviewees’ consent, two trained interviewers conducted the interviews and recorded them with a voice recorder to ensure the validity of the interview materials.

#### 3.4.2. Data Collection Procedures

Qualitative data were collected through interviews. The interviews were divided into three stages: (1) in-depth personal interviews were conducted with five community managers and five family doctors in the Z District who were responsible for digital health services for the elderly. The content of the interview comprised the mechanism of digital health services, its main methods, the design logic of the digital transformation of health services, and application effects and feedback of the transformation process. Each in-depth interview lasted 40 min to 1 h. (2) In-depth personal interviews were conducted with three service personnel and three volunteers in the Z District. The content of the interview was the participation mechanism, service content, and service effect of the service company. Each in-depth interview lasted 40 min to 1 h. (3) Focus group interviews with eight of the elderly in each of the three community groups in the Z District were conducted. The content of the interview was the perception, identity, and satisfaction of digital health care services. Each focus group interview lasted 90 min to 2 h. (4) Online in-depth personal interviews were conducted with four IT developers in the Z District using ZOOM software (Zoom Video Communications, Inc., San Jose, CA, USA). The interviews included the mechanism of digital health services, the technical requirements and development of the digital transformation of health services, and the application effects and feedback of the transformation process. Each in-depth interview lasted 40 min to 1 h.

#### 3.4.3. Data Analysis

(1) Data organization

The interview recordings and written data were organized into 110,000-word raw data. Two thirds of the contents were randomly selected for analysis and the other one third was used as a theoretical saturation test.

(2) Data extraction

The text data of the time series were extracted and the trajectory equifinality modeling method was used to analyze the logic path map of digital transformation based on the time series. The basic elements included the equifinality point (EFP), bifurcation point (BFP), obligatory passage point (OPP), irreversible time, and direction of social association. The EFP refers to different behaviors which tend to be directed toward the same goal, BFP means that different actions lead to different purposes, and OPP indicates the effects that the elderly must achieve as a result of policy or habit. Considering the differences in participants’ perceptions of the digital transformation of health services, this study sorted the logical path of digital transformation from the perspectives of the supply and demand sides.

(3) Data categorization

According to the initial concepts, categories and core categories were formed by coding and time series analysis, the relationship between categories was analyzed to form a storyline, and a digital health service aging transformation model was constructed. Open coding is an operational process of breaking up collected data, creating concepts, and then collating them in new ways. It is characterized by grouping the same phenomenon formed by different concepts and explained by high-level concepts. Based on this principle, we encoded the collected raw textual data word by word and conceptualized them step by step. Subsequently, we analyzed, compared, categorized, and interpreted the concepts. The categories generated by grounded theory open coding are independent and scattered; thus, the concrete relationship between categories must be further clarified. According to the logical relationship between the categories, axis coding summarizes the data to form the main category and subcategory, which is then reclassified. Selective coding requires unearthing the dominant core category from the main category and developing a storyline, encompassing most of the research findings within a broad theoretical scope, and validating relationships with known data.

(4) Grounded theory approach

Grounded theory requires testing as to whether the theory is saturated after the theory is constructed. Trajectory equifinality modeling was “inspired” by a grounded theory approach to verify data saturation. In this study, no new concepts and categories were found after analyzing one third of the original data.

This study was approved by the Waseda University Ethics Committee (REC number 2018-278) and Hangzhou Normal University (REC number 2021-1147). All participants were informed about the content of this study and signed informed consent. All study materials were stored in the laboratory of Hangzhou Normal University.

## 4. Results

### 4.1. Participants

A total of 156 people participated in this survey, which met the requirement of a sample size of at least 96. They included 60 men and 96 women; 135 people (86.5%) lived with partners, 16 people (10.2%) lived alone, and 5 people (3.3%) lived with children. Their mean age was 59.7 ± 19.2 years. Regarding educational background, 28 (17.9%) had completed primary school and below, 29 (18.6%) had completed junior high school, 40 (25.6%) had completed high school, and 59 (37.8%) had completed an undergraduate degree and above. Self-rated health was divided into unhealthy (including very unhealthy, unhealthy, and average) and healthy (healthy, very healthy). The number of unhealthy people was 42 (26.9%) and the number of healthy people was 114 (73.1%). Participants’ average perception score was 3.3 ± 0.5, identity was 3.5 ± 0.4, and satisfaction was 3.4 ± 0.6.

### 4.2. Differences in Perception, Identity, and Satisfaction under Digital Transformation

Univariate analysis showed that there were significant differences in the perception score of the supply and demand side, age, living conditions, and educational background (Table 2). The perception score of the supply side was higher than the demand side, a younger age was associated with a higher perception score, and the perception score of cohabitation with children was higher than the other situations. There were significant differences in the identity score of the supply and demand side, age, educational background, and self-rated health. The identity score of the supply side was higher than the demand side, a younger age was associated with a higher identity score, and self-rated healthy people’s identity score was lower than the unhealthy people. There were significant differences in the satisfaction score of the supply and demand side, age, educational background, and self-rated health. The satisfaction score of the supply side was higher than the demand side; the younger the age, the higher the satisfaction score, the higher the educational level, the higher the satisfaction score. Moreover, self-rated healthy people had lower satisfaction scores than unhealthy people.

### 4.3. Correlation Analyses of Perception, Identity, and Satisfaction

The correlation analyses showed significant positive relationships between perception, identity, and satisfaction on the supply side (Table 3). Perception (r = 0.48) and identity (r = 0.37) were moderately correlated with satisfaction. Significant positive relationships among perception, identity, and satisfaction on the demand side were also found. Perception was strongly correlated with identity (r = 0.69) and satisfaction (r = 0.64), and identity was strongly correlated with satisfaction (r = 0.56).

### 4.4. Influencing Factors in Perception, Identity, and Satisfaction

Regarding the perception of digital transformation, logistic regression analysis showed that the supply side scored 19.1 times higher than the demand side. Furthermore, the participants aged <40 years scored 116 times higher than the participants aged ≥60 years. The participants with university and above education scored 8.2 times higher than the participants with primary and below education. Regarding the identity of digital transformation, the supply side scored 5.4 times higher than the demand side. The participants aged <40 years scored 3.1 times higher than the participants aged ≥60 years. The participants with an unhealthy condition scored 2.3 times higher than the participants with a healthy condition. Regarding the satisfaction of digital transformation, the supply side scored 3.7 times higher than the demand side. The participants aged <40 years scored 11.2 times higher than the participants aged ≥60 years. The participants living with a partner scored 12 times higher than the participants living alone. The participants with university and above education scored 4.1 times higher than the participants with primary and below education (Table 4).

### 4.5. Qualitative Time Series Analysis

Based on the interview data, the logical path of digital transformation of time series was analyzed from the supply (government workers, social organization workers, IT developers) and demand side (the elderly) using the trajectory equifinality modeling method.

Among supply-side interviewees, 40 and 60% were male and female, respectively. The average age was 41.4 ± 10.2 years, and the working years were 10.6 ± 8.9 years (Table 5).

**Figure 4:**
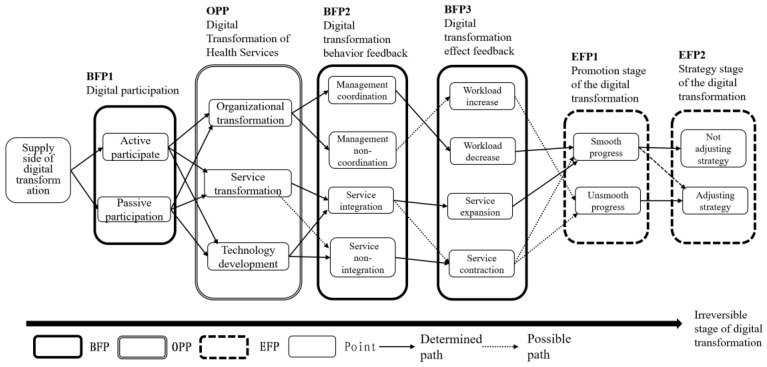
Time-series path of supply-side-based digital health service aging transformation.

During the digital transformation phase on the supply side (Figure 4), stakeholders tended to form two outcomes. The participation of government personnel and social organization staff in digital transformation was manifested as active participation and passive participation (BFP1). The digital transformation stage of health services consisted of three parts (OPP): organizational transformation, service transformation, and technology development. In the digital transformation behavior feedback stage (BFP2), management coordination/non-coordination of organizational transformation, service integration/non-integration of service transformation, and technology development were formed. In the digital transformation effect feedback stage (BFP3), workload increase/decrease was formed based on management behaviors and service expansion/contraction behaviors were formed based on service changes. In the promotion stage of the digital transformation of health services (EFP1), the results of smooth or unsmooth progress were formed. Finally, the behavior of not adjusting/adjusting the strategy was formed in the strategy stage of the digital transformation of health services (EFP2).

Participants indicated that how digital technology could meet the health needs of the elderly is a difficult factor in the technology development process. The main users of technology development are the supply side of health services for older people. Based on the recommendations of family doctors and volunteers, the government in Region Z procured wearable devices and distributed them to the elderly, and the function of the wearable devices was designed by the technology developers. In the process of collecting health data, although current wearable devices can collect data such as blood pressure, blood sample saturation, and heartbeat, the accuracy of the data is not as high as that of medical institutions.

Among the interviewees on the demand side, 41.7% were men and 58.3% were women. The average age was 72.1 ± 7.2 years, and the length of residence was 20.6 ± 7.9 years (Table 6).

**Figure 5:**
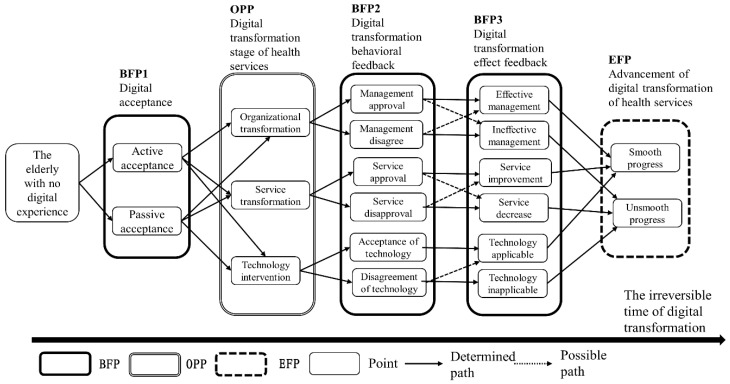
Time-series path of demand-side-based digital health service aging transformation.

In demand-side digital transformation (Figure 5), stakeholders tended to shape an outcome. The digital feedback of the elderly was active and passive acceptance (BFP1). With the advancement of the digital transformation stage of health services (OPP), organizational, service, and technical elements have changed synchronously, forming “management approval/disagreement of organizational transformation”, “service approval/disapproval of service transformation”, and “acceptance/disagreement of technology intervention” behavioral feedback (BFP2). The above content was converted into effect feedback (effective/ineffective management, service improvement/decrease, technology applicable/inapplicable) (BFP3). Ultimately, it was manifested as the smooth/unsmooth digital transformation of health services (EFP).

The elderly express that while they appreciate its importance, their inability to learn digital technology prevents them from adapting to digital transformation, and they need assistance from volunteers or family members to use digital health services. Also, they said that the existing digital health services were free, so they were willing to use them, but if payment was required, they may be reluctant to continue using the services.

### 4.6. Open Coding

Open coding is an operational process of breaking up collected data, creating concepts, and then putting them back together in new ways. It is characterized by grouping the same phenomenon formed by different concepts and explained by high-level concepts. Based on this principle, we encoded the collected raw textual data word by word and conceptualized them step by step. Subsequently, we analyzed, compared, categorized, and interpreted the concepts. A total of 668 original sentences and initial concepts were obtained. After eliminating invalid and repeated concepts, 125 valid concepts and 18 categories were obtained. Refer to Table 7 for the full list of categories and concepts.

### 4.7. Axial Coding

The categories generated by grounded theory open coding are independent and scattered; thus, the concrete relationship between categories must be further clarified. According to the logical relationship between the categories, axis coding summarizes the data to form the main category and subcategory, which is then reclassified. According to the above principles, this study extracted three main categories: “institutionalization of digital health service transformation”, “digital health service transformation service redesign”, and “digital health service transformation experience evaluation”. Refer to Table 8 for a full list of the main categories and subcategories.

### 4.8. Selective Coding

Selective coding requires unearthing the dominant core category from the main category and developing a storyline, encompassing most of the research findings within a broad theoretical scope, and validating relationships with known data. Based on repeated comparative analysis of the relationship between the main categories, the core of the case was summarized and extracted as “the dilemma and transformation path of health services for the elderly under digital transformation”. The story line around this core category was to optimize community public health services and respond to the “digital divide” of the elderly. The Hangzhou Z District has implemented an aging-appropriate transformation of digital health services. Faced with the following three questions in the transformation process: “1. What elements are the realistic dilemmas of digital health services for aging transformation?” “2. How does digital transformation intervene in the redesign of health services to meet the health needs of the elderly?” and “3. How can management and service institutions overcome the cognitive difficulties of the elderly and provide high-quality and convenient digital health services to this population?” the Z District has taken three measures: “institutionalization of digital health service transformation”, “digital health service transformation service redesign”, and “digital health service transformation experience evaluation”.

According to the analysis of raw data based on grounded theory, it was found that organizational, service, and technical elements affect the aging-appropriate transformation of digital health services from the macro, meso, and micro levels. Corresponding to the three main categories of ABC, we built a model for the aging transformation of digital health services.

### 4.9. Digital Transformation Model

**Figure 6:**
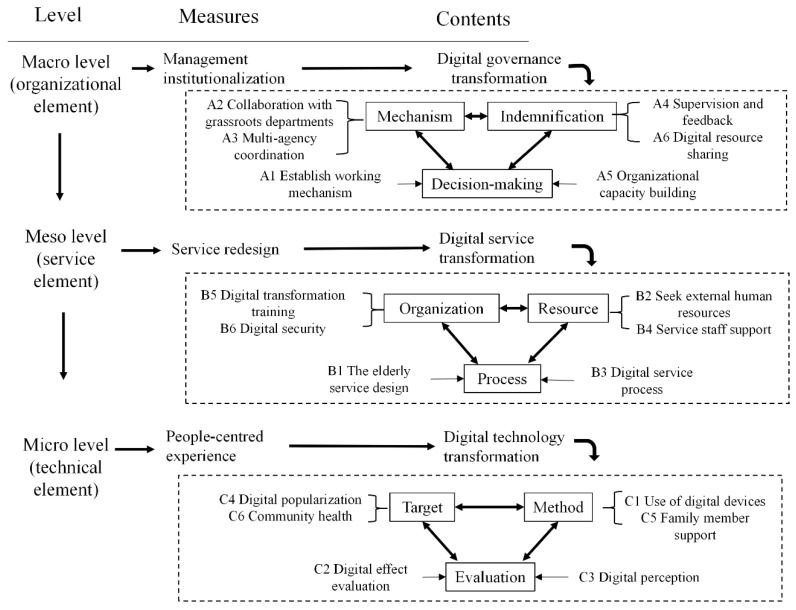
Age-appropriate transformation model of digital health services.

Based on the service ecology theory, an aging model of digital health service was constructed from the “macro–meso–micro” level (Figure 6). At the macro level, relying on organizational elements to promote the institutionalization of management, it included three categories: mechanism, indemnification, and decision-making. The meso-level relies on service elements to promote service design, including three categories: organization, resources, and process. At the micro level, relying on technical elements to implement “people-oriented” services, it includes three categories of target, methods, and evaluation.

## 5. Discussion

Based on the mixed research approach, this study explored the characteristics of digital health service transformation for the elderly and analyzed the behavioral differences of different stakeholders in the process of digital transformation. Results found that the sociological characteristics of the participants influenced their perceptions of digital transformation differently. When compared with the demand side, the supply side of digital services was more willing to actively participate in digital transformation. Based on the “macro–meso–micro” digital transformation model proposed in this case, the mechanism of digital transformation was explained from the management, service, and technology dimensions, being the main reason for the success of the community’s digital transformation.

The age-appropriate transformation of digital health services is the main path for the application of digital technology in the community. Participants had different perceptions of the age-appropriate transformation of digital health services and there were significant differences in the supply and demand side, age, education, and self-rated sense of health.

The results showed that the scores of the supply side in perception, identity, and satisfaction were significantly higher than those of the demand side. In terms of the perception score, the supply side was 19.1 times higher than the demand side, which is significantly higher than identity and satisfaction. The supply side is the subject of management and the provider of services and its sensitivity to digitalization is significantly higher than the demand side. In China, digital transformation guides service management through administrative decision-making rather than demand-oriented service management, which is why supply side scores may have been higher than the demand side [61]. Young people scored significantly higher than the elderly in perception, identity, and satisfaction and there was a significant difference in scores. The results of perception of those aged below 40 years were 116 times higher than those over 60, showing a hindrance of the “digital divide” in digital transformation. Due to the significant gap in perception, the difference in the identity and satisfaction between young people and the elderly is more obvious. The problem of the “digital divide” in the elderly is not only due to the barriers faced by digital technology [62], but also because existing digital technology fails to conform to the behavior habits of the elderly [63]. In solving the “digital divide” problem, the elderly’s perception of digital transformation should be improved, and, consequently, their identity and satisfaction should improve [64].

Educational background was the main influencing factor for the scores of digital transformation perception, identity, and satisfaction. The higher the education, the deeper the understanding of digital transformation and the higher the utilization of digital services. The main measure to improve the perception, identity, and satisfaction of less educated people in digital transformation is through community-based digital training [65]. The perception and identity of unhealthy people were significantly higher than healthy people. Unhealthy people’s demand for health knowledge is achieved through health services; thus, the digital transformation of health services should be extended to healthy people [66].

The supply side perception and satisfaction were moderately correlated. Furthermore, satisfaction and identity were moderately correlated. Moreover, the demand side’s perception, identity, and satisfaction were both positively and strongly correlated. The demand side accepts the continuous digital transformation of health services, which is the reason for the strong correlation between its perception, identity, and satisfaction [67]. However, the discontinuity among the supply side’s perception, identity, and satisfaction restrict the improvement of the service effect. Enhancing the continuity of service processes on the supply side can promote the continuous optimization of services [68].

Trajectory equifinality modeling revealed differences in perceptions between the supply and demand side during digital transformation. The supply side paid attention to management strategies in digital transformation and promoted digital transformation through the optimization of management mechanisms. China’s administrative management strategy is dominated by one-way management, resulting in government personnel managing projects after higher-level leadership decisions have been made; thus, it is difficult to introduce organizations outside the government in digital transformation [69]. The demand side paid more attention to the specific services of the elderly and gradually adjusted the digital transformation through the service perception of the elderly. This is a bottom-up governance path and gradually replaces the direct management of the government [70]. The weak learning ability of the elderly regarding digital transformation restricts the frequency of their digital technology use. Considering their cognitive decline, the development of digital technologies needs to be oriented toward the supply side [71]. The government needs to invest in digital health services for the elderly as free public services so that they can adapt to the digital society and be willing to use digital technology for health management [72].

The aging transformation of digital health services included governance, service, and technology transformation paths. These three paths relied on different content to promote digital transformation.

### 5.1. Digital Governance Transformation

Regarding mechanisms, a departmental coordination mechanism must be implemented. Civil affairs and health departments in the grassroots governance system work together to achieve management coverage to meet the diverse needs of the elderly. Sectoral coordination in the field of public health is the main measure of China’s health reform, and its successful results can be applied to the community [73]. Promoting the coordination of multiple institutions and linking the health services for the elderly to general hospitals from community health service institutions can be implemented. The orientation of health policy to the elderly is the main direction of governance transformation [74].

Regarding indemnification, monitoring and feedback paths must be established. Through the innovative designs of “setting up feedback mailboxes”, “contacting staff directly”, and “leadership reception day”, the elderly are given a path for supervision and feedback, thereby improving governance efficiency. Moreover, governance strategies need to be adjusted for different population characteristics [75]. Digital resource sharing integrates “personal information data” and “health data” into the community information database to ensure the traceability and security of information sources [76].

Regarding decision-making, establishing a working mechanism includes “establishing a digital department”, “clarifying project division of labor”, “regular meeting and discussion”, and “establishing a project team”. Regular tracking of the progress of the transformation and decisions made by the executive management is also required. China’s administrative decision-making is characterized by its rapidity, but decision-makers without health experience are prone to bias, which ultimately leads to a decline in the effectiveness of decision-making [77]. Organizational capacity building includes the regular development of capacity improvement projects such as “digital discussion meeting”, “organizational communication meeting”, “brainstorming”, and “expert consultation”. It aims to cultivate the digital thinking and practical ability of management and service personnel. This is also similar to the digital transformation cases in Europe [78].

### 5.2. Digital Service Transformation

On the organizational side, digital transformation training refers to regular training in digital skills for service workers and clients. The training focuses on digital skills, thus, laying the foundation for the popularization of digital applications [79]. Regarding digital safety and security, paying attention to the elderly through infrared smoke sensor technology has been shown to be an effective measure to reduce the safety risks of the elderly [80].

Regarding resources, seeking external forces includes volunteer participation, company promotion, and institutional preferential services. The input of external forces has effectively alleviated the shortage of staff and brought a new situation of intervention for traditional services. To illustrate, volunteering as a supplement to human resources has been widely used in Norway [81]. Service personnel assistance includes registration guidance, medical reminders, department guidance, and electronic equipment guidance, which are reflected in the window service of health institutions. The complementarity of human and digital resources is a necessary measure for digital transformation [82].

Regarding processes, the experience of the elderly is the main direction of the service design for the elderly [83]. The aging-friendly service design includes the redesign of hardware and software, such as font enlargement, slowing down the process, and notification sound amplification. It is an aging-friendly transformation of traditional medical and health services. The digital service process includes the use of mobile phones throughout the process, paperless services, telemedicine, electronic health codes, electronic medical insurance cards, and electronic health records. It is a functional copy of upgrading traditional services to online services. In this stage of digital transformation, the acceptability of the demand side must be considered, otherwise the supply and demand sides are prone to be misaligned [84].

### 5.3. Digital Technology Transformation

Regarding the target, the popularization and promotion of digitalization is the goal proposed from the overall governance level. Community staff should actively connect with the elderly, provide digital tools, improve their digital health literacy, and integrate the elderly into the digital society [85]. Community health is the goal of the digital transformation of health care. The improvement of digital medical service capabilities is the main measure to comprehensively improve the health of the elderly [86].

Regarding methods, the use of digital devices includes software use, represented by health applications, and hardware use, represented by an automatic registration machine. The use of this type of equipment is linked to the transformation of aging-appropriate services, providing full-process digital medical services for the elderly. The age-appropriate design of digital devices reduces the sense of discomfort of the elderly in the digital society [87]. Family member support serves as a digital education guide for the elderly. Relying solely on volunteers or the human resources of health services cannot provide continuous support to the elderly; thus, family members fill this gap and support them to adapt to the digital society [88].

Regarding evaluation, the evaluation of digital effects includes the regular implementation of service satisfaction and surveys for the elderly, which can illuminate the reasons for any difficulties of digital health services for the aging transformation, and digital strategies can be adjusted accordingly [89]. The digital perception experience refers to the behavioral tendency after digital transformation. The elderly prefer to go to community hospitals rather than general hospitals. This is because it has become a unique phenomenon in China that the experience of digital health services in the community is stronger than that in general hospitals [90].

From the perspective of policy optimization, policy design can be carried out from the following four aspects:

1. Form an institutionalized decision-making process for management, consolidate the premise and foundation of digital transformation from the organization and security content, and realize governance transformation by relying on the establishment of working mechanisms and organizational capacity building.
2. Focus on service redesign, recruit volunteers to help consolidate human resources, and realize the service transformation of aging-appropriate design on the process side.
3. Assess the possible effects of the integration of digital technology on the elderly population and obtain feedback to achieve technological transformation through digital device use and family member support.
4. Digital transformation should consider irreversible time changes, and it is necessary to track behavioral and effect feedback in the process to design more effective strategies.

According to qualitative and quantitative research, the digital transformation results of health services for the elderly reflect the difference in perception and evaluation of digital transformation between the supply and demand side, and gradually change the health behavior of both. Different perceptions and evaluations require digital transformation to give priority to the supply side. The health level of the elderly can be improved only through supply-side digital health services [91]. Concurrently, the demand side puts forward requirements and developers implement the design of digital technology. The supply side needs to regularly ask the elderly about the health needs of the mechanism to continuously optimize the digital transformation [92]. In the process of digital transformation, different digital health services need to be designed considering their varied impact on the health behavior of the supply and the demand side [93].

### 5.4. Limitations and Future Research

There are some limitations in this study. This study was limited to the data of a single area in Hangzhou City; thus, the generalizability of the findings is lacking. In this study, the supply side of digital health services was mainly provided by government agencies, and the digital health services provided by enterprises were not reflected in the text. In future research, we will continue to carry out a combination of quantitative and qualitative research based on this study to analyze the action strategies and influencing factors of the aging-appropriate transformation of digital health services more accurately. We will provide wearable devices for the elderly, track their health data, and analyze the changes in their health indicators after using digital technology.

## 6. Conclusions

This study suggests that perception, identity, and satisfaction in the process of digital transformation are affected by factors such as the supply and demand side, age, education, and self-rated health. Digital transformation must consider factors such as age and learning ability of the elderly. Macro level management institutionalization, meso level service redesign, and micro level people-orientation build a service ecosystem for digital transformation and promote governance, service, and technology transformation. The application of the service ecosystem to digital transformation explains the following: (1) digital technology is an operational resource that can act on multi-level resources to realize value co-creation. It not only changes the behavior of users at the micro level, but also reconstructs the interaction and connection between service design at the meso level and institutionalization at the macro level. (2) Age-appropriate transformation must consider the experiences of the elderly and integrate different resources into macro-governance and meso-services. (3) The design of public health services must be people-centered and fully grasp the user experience. Overall, this study deepened the understanding of macro-organizational elements, meso-service elements, and micro-technical elements of digital transformation practices. The results have positive significance for promoting local health and related administrative agencies to take appropriate action strategies and improve the effect of digital transformation.

## Figures and Tables

**Table 1:** Results of factor analysis after rotation.

Item	1	2	3
Perception of organization	0.706		
Perception of service	0.682		
Perception of technology	0.677		
Identity of organization		0.676	
Identity of service		0.663	
Identity of technology		0.641	
Satisfaction of organization			0.586
Satisfaction of service			0.570
Satisfaction of technology			0.522

**Table 2:** Univariate analysis of perception, identity, and satisfaction.

Items	Perception	*p*	Identity	*p*	Satisfaction	*p*
Supply or demand side		<0.01		<0.01		<0.01
Supply side	3.8 ± 0.4		3.7 ± 0.4		3.7 ± 0.4	
Demand side	3.1 ± 0.4		3.3 ± 0.5		3.4 ± 0.4	
Gender		0.58		0.26		0.64
Male	3.5 ± 0.5		3.5 ± 0.6		3.5 ± 0.5	
Female	3.4 ± 0.5		3.5 ± 0.5		3.4 ± 0.5	
Age		<0.01		<0.01		<0.01
<40	4.0 ± 0.2		3.8 ± 0.3		3.8 ± 0.3	
40–59	3.7 ± 0.4		3.6 ± 0.4		3.5 ± 0.6	
≥60	3.2 ± 0.4		3.3 ± 0.5		3.4 ± 0.5	
Residence		0.04		0.62		0.05
Living with partner	3.4 ± 0.5		3.5 ± 0.4		3.5 ± 0.4	
Living with children	3.7 ± 0.5		3.6 ± 0.5		3.7 ± 0.4	
Living alone	3.2 ± 0.4		3.4 ± 0.5		3.2 ± 0.4	
Education		<0.01		0.01		0.02
Primary and below	3.3 ± 0.4		3.4 ± 0.5		3.3 ± 0.4	
Junior	3.2 ± 0.3		3.3 ± 0.4		3.4 ± 0.5	
High	3.3 ± 0.5		3.4 ± 0.6		3.4 ± 0.5	
University and above	3.8 ± 0.4		3.7 ± 0.4		3.7 ± 0.4	
Self-rated health		0.13		<0.01		0.03
Healthy	3.2 ± 0.5		3.3 ± 0.6		3.4 ± 0.4	
Unhealthy	3.5 ± 0.5		3.6 ± 0.5		3.5 ± 0.5	

**Table 3:** Correlation analyses of perception, identity, and satisfaction.

Supply Side				Demand Side			
	Perception	Identity	Satisfaction		Perception	Identity	Satisfaction
Perception	1			Perception	1		
Identity	0.24	1		Identity	0.69 **	1	
Satisfaction	0.48 **	0.37 **	1	Satisfaction	0.64 **	0.56 **	1

** *p* < 0.01.

**Table 4:** The logistic regression analysis of perception, identity, and satisfaction.

	Perception OR (95%CI)	*p*	Identity OR (95%CI)	*p*	Satisfaction OR (95%CI)	*p*
Supply or demand side						
Supply side	1		1		1	
Demand side	19.1 (8.7–45.1)	<0.01	5.4 (2.6–10.9)	<0.01	3.7 (1.8–7.2)	<0.01
Age						
≥60	1		1		1	
40–59	10.4 (4.2–25.1)	<0.01	3.9 (1.7–8.9)	<0.01	1.9 (0.8–4.2)	0.09
<40	116 (14.7–909.5)	<0.01	8.6 (3.0–24.7)	<0.01	11.2 (3.6–34.9)	<0.01
Residence						
Living alone	1		1		1	
Living with partner	3.2 (0.3–30.2)	0.08	1.6 (0.2–10.2)	0.06	3.7 (0.4–34.1)	0.24
Living with children	8.8 (0.7–99.2)	0.29	2.5 (0.3–19.5)	0.38	12 (1.1–141.3)	0.04
Education						
Primary and below	1	1			1	
Junior	0.4 (0.1–1.5)	0.85	0.6 (0.2–1.7)	0.36	1.2 (0.4–3.7)	0.66
High	0.9 (0.3–2.5)	0.19	0.8 (0.3–2.2)	0.74	1.1 (0.4–2.9)	0.88
University and above	8.2 (2.9–22.8)	<0.01	3.1 (1.2–7.9)	0.02	4.1 (1.5–10.6)	<0.01
Self-rated health						
Healthy	1		1		1	
Unhealthy	3.9 (1.7–8.8)	<0.01	2.3 (1.1–4.7)	0.02	0.6 (0.3–1.2)	0.15

**Table 5:** Supply-side interviewer information.

No.	Job	Gender	Age	Working Years
1	Community manager	Female	40	10
2	Community manager	Female	28	5
3	Community manager	Female	55	36
4	Community manager	Female	42	14
5	Community manager	Male	30	6
6	Family doctor	Male	45	18
7	Family doctor	Female	40	16
8	Family doctor	Male	36	8
9	Family doctor	Female	52	26
10	Family doctor	Female	47	23
11	Service personnel	Male	44	6
12	Service personnel	Female	32	2
13	Service personnel	Female	56	6
14	Volunteer	Female	56	6
15	Volunteer	Female	55	5
16	Volunteer	Female	50	2
17	IT developer	Male	27	5
18	IT developer	Male	34	8
19	IT developer	Male	30	6
20	IT developer	Male	30	5

**Table 6:** Demand-side interviewer information.

No.	Gender	Age	Years of Residence	Utilization of Digital Health Services
1	Male	62	22	Telehealth, Electronic health monitoring
2	Female	63	14	Online health consultation
3	Male	82	34	Wearable devices, Online health consultation
4	Female	71	28	Telehealth
5	Female	72	22	Telehealth, Wearable devices
6	Female	73	28	Online health consultation
7	Female	62	6	Online health consultation
8	Male	82	32	Telehealth
9	Male	77	24	Medication reminder, Online health education
10	Female	72	25	Online health consultation
11	Female	78	15	Wearable devices, Electronic health monitoring
12	Female	67	26	Online health education
13	Male	81	13	Telehealth
14	Male	80	17	Online health consultation
15	Male	67	22	Wearable devices, Online health education
16	Female	63	10	Online psychological consultation
17	Female	65	9	Telehealth
18	Male	63	20	Online health consultation, Medication reminder
19	Male	75	12	Online health education
20	Female	81	32	Online health education
21	Male	81	27	Online health consultation
22	Female	65	11	Telehealth, Wearable devices
23	Female	76	25	Online health consultation
24	Female	72	22	Online health consultation

**Table 7:** Concepts and categories formed by open coding.

Categories	Concepts
Establish working mechanism	Establish digital teams, clarify project division, establish an information reporting system, regular meetings, establish project teams, select young managers, establish an analysis system, determine work procedures.
Collaboration with grassroots departments	Multi-community collaboration, community meeting room sharing, building management cooperation, service experience sharing, unified security management, service group notification, vaccination records.
Multi-agency coordination	Two-way referral service, appointment registration, nurse communication, the elderly health service coordination, family doctor team, welfare supplies on behalf of others, drug distribution, the elderly housekeeping services.
Supervision and feedback	Set up feedback mailbox, work progress report, confirm partner authority, information release review, service effect evaluation, service content feedback, leadership reception day.
Organizational capacity building	Digital discussion meeting, organizational communication meeting, digital thinking, brainstorming, project discussion, Dingding App daily report, WeChat App operation, expert consultation.
Digital resource sharing	Data sharing, data backup, data traceability, community information registration, SMS reminder, service record synchronization, information covering the whole community.
The elderly service design	Enlarge fonts, slow down processes, amplify notification sounds, health and wellness knowledge, free health lectures, traditional Chinese medicine services, regular telephone calls.
Seek external human resources	Volunteer participation, college students caring for the elderly, provision of sphygmomanometer, public welfare promotion, business preferential services.
Digital service process	Use mobile phones throughout the process, paperless, telemedicine, Dingding video, QR code service, electronic health code, electronic medical insurance card, smart registration, electronic health record.
Service staff support	Guidance for appointment registration, medical reminder, department guidance, electronic signboard, electronic questionnaire, electronic equipment guidance.
Digital transformation training	Digital training, development of new digital functions, daily Dingding report, entry of electronic information records, mobile phone training for the elderly.
Digital security	Risk control, personal information privacy, information collection protocol, electronic police, infrared smoke sensor, focus on key groups, prevention of telecommunication fraud.
Use of digital devices	Use of registration APP, use of self-service registration machines, wearing of smart wristbands, electronic test list printer, electronic triage, electronic hospital guidance.
Digital effect evaluation	Decreased medical satisfaction, decreased medical time, increased risk, difficulty with electronic use, insufficient health reminders.
Digital perception	Willing to go to a community health service center, unwilling to go to a general hospital, weak experience, not suitable for the elderly, complex digital operations.
Digital popularization	Door-to-door support from social workers, distribution of mobile phones for the elderly, telephone notification for the elderly, registration to receive gifts.
Family member support	Electronic family network, family member early warning notice, family member teaching, family member accompanying medical treatment.
Community health	Chronic diseases, traditional Chinese medicine, vaccination, epidemic prevention and control, first aid measures, AED first aid.

**Table 8:** The categories and relational connotations of the axial coding.

Main Categories	Subcategories	Concept Explanation
A. Institutionalization of digital health service transformation	A1 Establish working mechanism	Establishing a working mechanism is the guarantee of digital transformation
	A2 Collaboration with grassroots departments	Collaboration between grassroots departments is the internal consensus of digital transformation
	A3 Multi-agency coordination	Multi-agency coordination is the premise to meet the diverse health needs of the elderly
	A4 Supervision and feedback	Monitoring and feedback ensure that the organization’s risks can be controlled
	A5 Organizational capacity building	Organizational capacity building is the internal driving force for digital transformation
	A6 Digital resource sharing	Digital resource sharing is the data foundation for digital transformation
B. Digital health service transformation service redesign	B1 The elderly service design	Age-friendly service design is the goal of digital transformation
	B2 Seek external human resources	External resources can expand digital transformation resources
	B3 Digital service process	Digital service process is a direct manifestation of digital transformation
	B4 Service staff support	Service personnel promote humanistic care under digital transformation
	B5 Digital transformation training	Digital transformation training enhances digital capabilities at different stages
	B6 Digital security	Digital security is the premise of digital transformation
C. Digital health service transformation experience evaluation	C1 Use of digital devices	Digital device usage is an enabling tool for digital transformation
	C2 Digital effect evaluation	Digital effect evaluation reflects the recognition of the elderly
	C3 Digital perception	Digital perception experience is the source of service optimization
	C4 Digital popularization	Digital popularization can expand the value of digital transformation
	C5 Family member support	Family member support is a family requirement for the elderly to embrace digital
	C6 Community health	Community health is final result of digital transformation

## Data Availability

Data are available from the authors at reasonable written request after authorization by the Data Protection Office of the School of Public Health, Hangzhou Normal University, China.
